# Surface waves on a soft viscoelastic layer produced by an oscillating microbubble

**DOI:** 10.1039/c5sm03084f

**Published:** 2016-04-07

**Authors:** Marc Tinguely, Matthew G. Hennessy, Angelo Pommella, Omar K. Matar, Valeria Garbin

**Affiliations:** a Department of Chemical Engineering , Imperial College London , London SW7 2AZ , UK . Email: v.garbin@imperial.ac.uk

## Abstract

Ultrasound-driven bubbles can cause significant deformation of soft viscoelastic layers, for instance in surface cleaning and biomedical applications. The effect of the viscoelastic properties of a boundary on the bubble–boundary interaction has been explored only qualitatively, and remains poorly understood. We investigate the dynamic deformation of a viscoelastic layer induced by the volumetric oscillations of an ultrasound-driven microbubble. High-speed video microscopy is used to observe the deformation produced by a bubble oscillating at 17–20 kHz in contact with the surface of a hydrogel. The localised oscillating pressure applied by the bubble generates surface elastic (Rayleigh) waves on the gel, characterised by elliptical particle trajectories. The tilt angle of the elliptical trajectories varies with increasing distance from the bubble. Unexpectedly, the direction of rotation of the surface elements on the elliptical trajectories shifts from prograde to retrograde at a distance from the bubble that depends on the viscoelastic properties of the gel. To explain these behaviours, we develop a simple three-dimensional model for the deformation of a viscoelastic solid by a localised oscillating force. By using as input for the model the values of the shear modulus obtained from the propagation velocity of the Rayleigh waves, we find good qualitative agreement with the experimental observations.

## Abstract 2

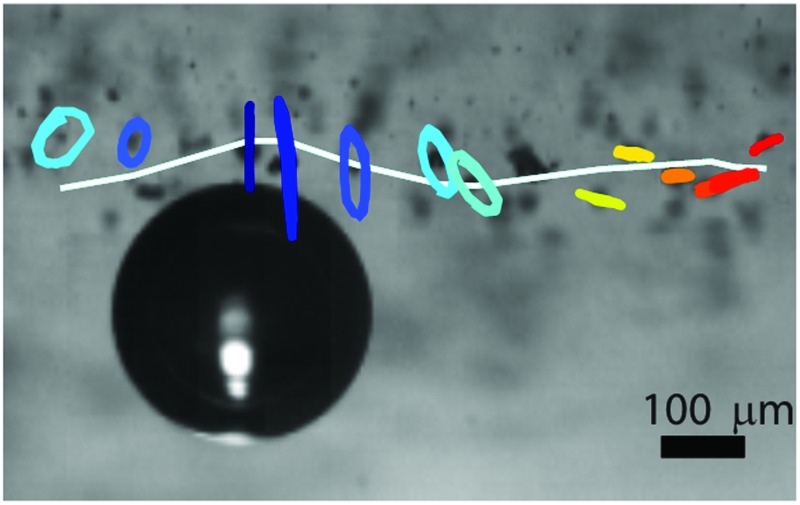
An ultrasound-driven microbubble undergoing volumetric oscillations deforms a soft viscoelastic layer causing propagation of a surface elastic wave. High-speed video microscopy reveals characteristics of the elliptical particle trajectories that depend on the rheological properties of the layer.

## 1 Introduction

Cavitation and bubble dynamics near solid boundaries have been investigated extensively in the context of underwater acoustics and surface cleaning,^1^ and more recently for biomedical applications.^2^ The proximity of a solid boundary affects the dynamic response of microbubbles in ultrasound^3–6^ and causes hydrodynamical phenomena such as microstreaming^7^ and microjetting,^8^ which can deform and disrupt biomaterials.^9–11^ Theoretical models and numerical simulations of the effect of the proximity of a deformable boundary on bubble dynamics predict a shift in resonance frequency of a bubble near a compliant boundary,^12,13^ a change in nonlinear response,^14^ and microjet formation.^15,16^ However, the existing models do not always capture the strong dependence of these phenomena on the mechanical properties of the boundary, and some issues remain open. For instance, two models currently available to describe the dynamics of an ultrasound-driven microbubble near a compliant boundary^12,13^ predict resonance frequency shifts in opposite directions for interfaces with the same mechanical properties and, when compared directly with experimental measurements,^5,6^ they provide conflicting results. These discrepancies call for a better fundamental understanding of the interaction of oscillating bubbles with soft, deformable boundaries. The available experimental measurements are limited to the case of bubble collapse, possibly accompanied by shock-wave emission, and jet formation.^17–19^ In contrast, the dynamic deformation of a viscoelastic boundary induced by the volumetric oscillations of an ultrasound-driven bubble has not been investigated so far.

The response of viscoelastic layers to oscillatory deformation has been studied either by using oscillating actuators,^20–22^ or electric fields.^23^ The applied oscillatory deformation excites surface waves, which have typically been recorded by light scattering or interferometric techniques. Hydrogels, for instance agarose gels, are often used as model systems for soft viscoelastic layers, because the mechanical properties can be tuned by changing the gel concentration or by using different molecular weights.^24^ Experiments on surface wave propagation on agarose gels have revealed the existence of two dynamic regimes:^20,21,23^ at sufficiently low frequency of deformation, the elastic properties of the material dominate, and the propagating wave is an elastic wave, or Rayleigh wave.^25,26^ For higher frequencies, the viscoelastic layer behaves predominantly as a fluid, and the propagating wave becomes a capillary wave. The transition from elastic to capillary waves occurs when the frequency of the oscillating deformation exceeds a crossover frequency, *f*_c_, given by:^20^
1
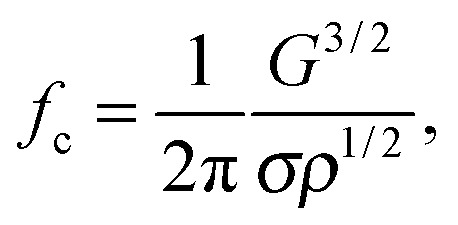
where *G* is the shear modulus of the gel, *ρ* its density, and *σ* the surface tension. Measurements of Rayleigh wave propagation have enabled the measurement of the frequency-dependent viscoelastic properties of soft layers.^22^ A crossover frequency has been predicted to exist also for Rayleigh wave propagation on nematic elastomers, marking a transition from liquid crystalline behaviour to solid-like elastic behaviour.^27^ Previous experimental work on surface wave propagation on agarose gels has relied on measurements of the amplitude decay and the propagation speed, but has not revealed the microscopic details of the surface deformation. The kinematics of deformation of the surface, particularly in the vicinity of the source of applied stress, is indeed one of the outstanding questions on the interaction of oscillating bubbles with soft, deformable boundaries.

In this paper, we measure the dynamic deformation of an agarose gel layer induced by the linear oscillations of ultrasound-driven microbubbles. The dynamics of single bubbles in contact with the surface of the gel, and the dynamic deformation of the surface, are recorded using high-speed video microscopy. To overcome the limitations of previous studies, we perform direct visualisation of the displacement of the gel surface, by tracking the trajectories of embedded tracer particles. Using this method we have access not only to the surface wave propagation speed and amplitude decay, but also to the time-resolved particle displacements as a function of distance from the bubble, and as a function of time. We study the effect of the rheological properties of the layer by tuning the agarose gel composition. We first present the experimental results for the deformation field in the gel induced by the bubble motion. A model for the deformation of a viscoelastic solid is then developed, and predictions of the model for conditions similar to those of the experiment are presented. Finally, the predictions of the model are compared with the experimental results.

## 2 Materials and methods

### 2.1 Gel preparation and characterization

We produced agarose gels with controlled viscoelastic properties by tuning the gel concentration. We present results for four concentrations, 0.5%, 1%, 2% and 5% w/v. Agarose powder (A9539, Sigma Aldrich) is mixed with ultrapure water (resistivity 18.2 MΩ cm; Milli-Q filtration system, Millipore) at room temperature. The solution is heated to 95 °C and stirred for 30 minutes, then poured into a container, and left to set at room temperature to form layers with a thickness of 7 mm. Tracer particles for particle image velocimetry (9–13 μm hollow glass spheres, 110P8, LaVision GmbH) are immediately spread on the surface of the solution so that they become embedded in a thin superficial layer as the solution forms a gel upon cooling to room temperature. The spreading solution for particle deposition is a 1 : 1 mixture of water and isopropyl alcohol, and evaporates sufficiently quickly that the effect on the gel properties is negligible. We measured the rheological properties using a stress-controlled rotational rheometer (Discovery HR-1, TA Instruments), with a plate–plate geometry. The shear modulus, *G*, was measured from a creep test using a constant stress of 100 Pa. The storage and loss moduli, *G*′ and *G*′′, respectively, were measured at a frequency of 10 Hz. The data presented for *G*′ and *G*′′ correspond to the average value measured over stresses in the ranges 0.1–10 Pa for gels with 0.5% w/v concentration, 1–100 Pa for gels with 1% and 2% w/v concentration, and 10–1000 Pa for gels with 5% w/v concentration. The results are summarised in Table 1.

**Table 1:** Viscoelastic properties of agarose gels with 0.5%, 1%, 2% and 5% w/v concentration, measured by creep test and oscillatory rheometry

Gel concentration (w/v)	0.5%	1%	2%	5%
*G* from creep test (kPa)	1.4 ± 0.2	4.7 ± 0.1	43 ± 2	318 ± 10
*G*′ at 10 Hz (kPa)	1.52 ± 0.02	5.73 ± 0.01	49.3 ± 0.3	358 ± 2
*G*′′ at 10 Hz (kPa)	0.08 ± 0.01	0.26 ± 0.02	1.6 ± 0.3	12 ± 2

### 2.2 Experimental setup and methods

**Fig. 1:**
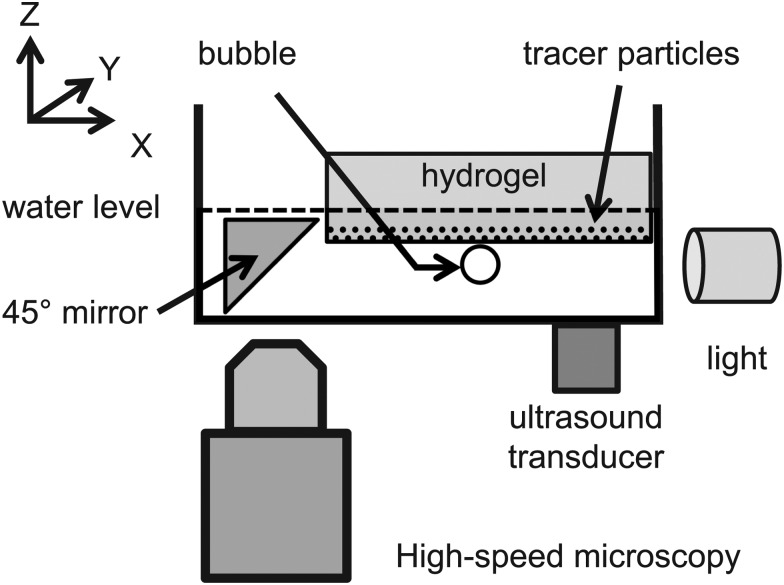
Schematic diagram of the experimental setup and definition of coordinate system. The surface of the gel lies in the (*x*,*y*) plane. The vertical axis *z* points into the gel layer.

The experimental setup is shown in Fig. 1. A single-element piezoelectric transducer (Physik Instrumente, P-121.05, resonance frequency 100 kHz) was glued on the bottom surface of a glass container positioned on the stage of an inverted microscope (Olympus, IX71). A gel sample (12 mm wide, 24 mm long, and 7 mm thick) was positioned on two spacers made of elastomer, and the container was filled with ultrapure water so as to submerge the bottom surface of the gel. A 45° mirror was placed in the water for side-view visualisations through the microscope objective located below the dish. By moving the microscope stage, the objective can be positioned either below the mirror or directly below the bubble, for side-view or bottom-view imaging, respectively. The bubble was illuminated in transmission mode. For side-view imaging a fibre light source (Schott, KL 2500 LED) was used. The dynamics of the bubble and the deformation of the gel were imaged at 4× magnification and recorded using a high-speed camera (Photron, FASTCAM SA5) at 210 000 frames per second. The piezoelectric transducer was actuated by the signal produced by a waveform generator (Agilent, 33220A), and amplified by a linear radio-frequency power amplifier (T&C Power Conversion, AG 1021). The amplitude of the pressure fluctuations in water was recorded with a hydrophone (RP acoustics, PVDF RP 33 s).

Microbubbles of controlled size were generated with a co-flowing microfluidic device.^28^ Nitrogen flowed through the inner capillary (TPS100200, CM Scientific, inner diameter 100 μm), while water flowed through the outer one (8290, CM Scientific, inner diameter 900 μm). The size of the microbubbles was controlled by adjusting the gas and water flow rates. Microbubbles injected below the gel rise to its bottom surface by buoyancy forces. All but one of the microbubbles were then moved away from the observation zone using a glass capillary. For the experiments presented here, we produced sets of monodisperse bubbles. The bubbles used with the 0.5% gel had a radius *a* = (180 ± 5) μm, and the oscillations were induced *via* ultrasound forcing at 18 kHz. The bubbles used with the 1% gel had a radius *a* = (190 ± 5) μm, and the oscillations were induced at 17 kHz. The bubbles used with the 2% and 5% gel had a radius of *a* = (160 ± 5) μm, and the oscillations were induced at 20 kHz.

The ultrasound frequency is set so as to match the resonance frequency of the bubble, given by Minnaert's formula, 
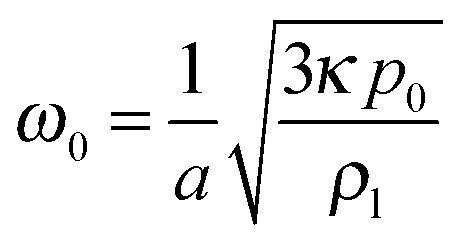
, with *p*_0_ the hydrostatic pressure, *κ* the polytropic exponent of the gas, and *ρ*_l_ the liquid density.^29^ For each measurement, a single ultrasound burst of 5 cycles was applied. The peak-to-peak acoustic pressure amplitude was (42 ± 5) kPa for the 0.5%, 1% and 2% gels, and (105 ± 14) kPa for the 5% gel, resulting in a maximum amplitude of bubble oscillations between 3% and 9% of the initial bubble radius. Under these conditions, the bubble oscillations remained approximately spherical, with only a small deviation due to the proximity of the elastic boundary: the deformation parameter *D* = |*d*_1_ – *d*_2_|/(*d*_1_ + *d*_2_), where *d*_1_ is the major axis and *d*_2_ the minor axis, had a maximum value *D* ≈ 3% for all concentrations used. We also tracked the vertical position of the contact point between the bubble and the gel surface as a function of time, and confirmed through Fourier mode decomposition that the only frequency component corresponds to the driving frequency of 18 kHz, 17 kHz, and 20 kHz, respectively. When a bubble oscillates near a boundary, it is attracted towards rigid boundaries, and repelled by soft boundaries due to the interaction of the ultrasound wave scattered by the bubble with the boundary.^1^ In our experiments we observe that the bubbles translate in the direction normal to the gel surface, with a maximum displacement of the center of mass of 1 μm away from the 0.5% and 1% gel, 3 μm away from the 2% gel, and 25 μm towards the 5% gel. By investigating both the cases of attraction and repulsion we confirmed that the small displacement of the center of the bubble does not significantly affect the observed phenomena.

### 2.3 Particle tracking

The deformation of the gels is measured by tracking the displacement of the embedded tracer particles. The position of the particles in each frame is determined with sub-pixel resolution by image processing in two steps. In the first step, regions of interest are manually defined in the first frame of the movie, centred around the particles that are in the same focal plane as the bubble. The movie is then converted to binary scale, and the location of each particle within the region of interest is extracted for each frame by finding the local maxima on the binary image. This step gives the positions of the particles with pixel accuracy. To improve the accuracy, in the second step the brightness of the pixels in the original images is used. For each particle and each frame of the movie, the value of the brightness of the pixels is set to zero everywhere on the image, except for a disk of radius of 3 pixels centred at the location of the particle calculated in the first step. The center of mass of the particle is calculated using the brightness of the pixels as weight. This step gives sub-pixel accuracy with an uncertainty of 0.1 pixel.^30^

## 3 Experimental results

**Fig. 2:**
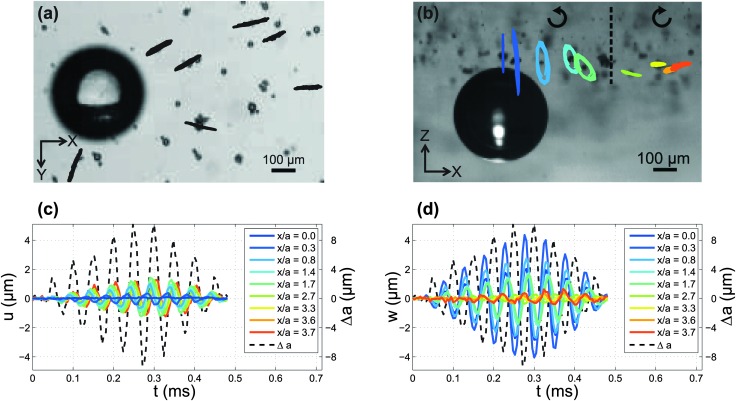
(a) Trajectories of tracer particles from a bottom-view high-speed movie of a 2% gel. The displacement is magnified by a factor of 50 for clarity. (b) Trajectories of tracer particles from a side-view high-speed movie of a 2% gel. The displacement is magnified by a factor of 25. The particles move along elliptic trajectories in a direction indicated by the arrows: prograde direction near the bubble, retrograde direction away from the bubble. (c) Temporal evolution of the horizontal particle displacement *u* for particles at different distances from the oscillating bubble. (d) Temporal evolution of the vertical particle displacement *w*. The dashed lines in (c) and (d) represent the radial excursion of the bubble, Δ*a*.

High-speed imaging and particle tracking reveal the kinematics of deformation of the gel due to the ultrasound-induced microbubble oscillations. The pressure on the gel is high (low) when the bubble expands (is compressed). The resulting deformation of the gel over several cycles of oscillations is shown in Fig. 2 for a 2% gel. The bottom-view visualisation in the (*x*,*y*) plane [Fig. 2(a)] shows that the trajectories of the particles are radial from the center of the bubble. The side-view visualisations in the (*x*,*z*) plane [Fig. 2(b)] reveal a complex behaviour of the particle displacement. The tracer particles follow elliptical trajectories, consistent with the propagation of surface elastic waves, or Rayleigh waves.^25^ Interestingly, the direction of rotation of the particles along the elliptic trajectories is prograde in the vicinity of the bubble, and shifts to retrograde at a certain distance from the bubble. Furthermore, the tilt angle *θ* of the major axis of the ellipses relative to the vertical direction, changes with the distance from the bubble. Fig. 2(c and d) show the temporal evolution of the horizontal displacement, *u*, the vertical displacement, *w*, and the radial excursion of the bubble, Δ*a*. Both components of the displacement oscillate at the frequency of the applied deformation. This observation indicates that the gel layer is sufficiently thick so as to not confine the particle displacement. Such a confinement could result in the propagation of multiple modes.^31^ Control experiments confirm that the interface deformation upon propagation of the ultrasound wave, but without a bubble on the surface, is negligible.

**Fig. 3:**
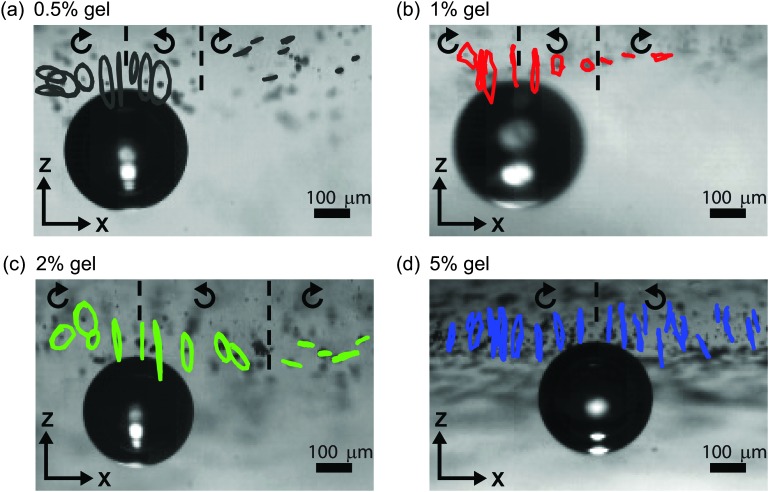
Trajectories of tracer particles from a side-view high-speed movie for (a) 0.5% gel, (b) 1% gel, (c) 2% gel, and (d) 5% gel. The displacement is magnified for clarity by a factor of 10, 40, 20, and 30, respectively. The arrows indicate the direction of motion of the particles.

Fig. 3 compares qualitatively the kinematics of deformation for the four gels (0.5%, 1%, 2%, and 5% concentration). The particle trajectories are elliptical for all four gels, with the amplitude decay and the variation of the tilt angle depending strongly on the rheological properties of the gel. The shift from prograde to retrograde rotation occurs farther from the bubble for stiffer gels. The transition occurs at *x*/*a* = 1.2 for the 0.5% gel, *x*/*a* = 1.3 for the 1% gel, at *x*/*a* = 2.2 for the 2% gel, and at *x*/*a* = 4.3 for the 5% gel.

**Fig. 4:**
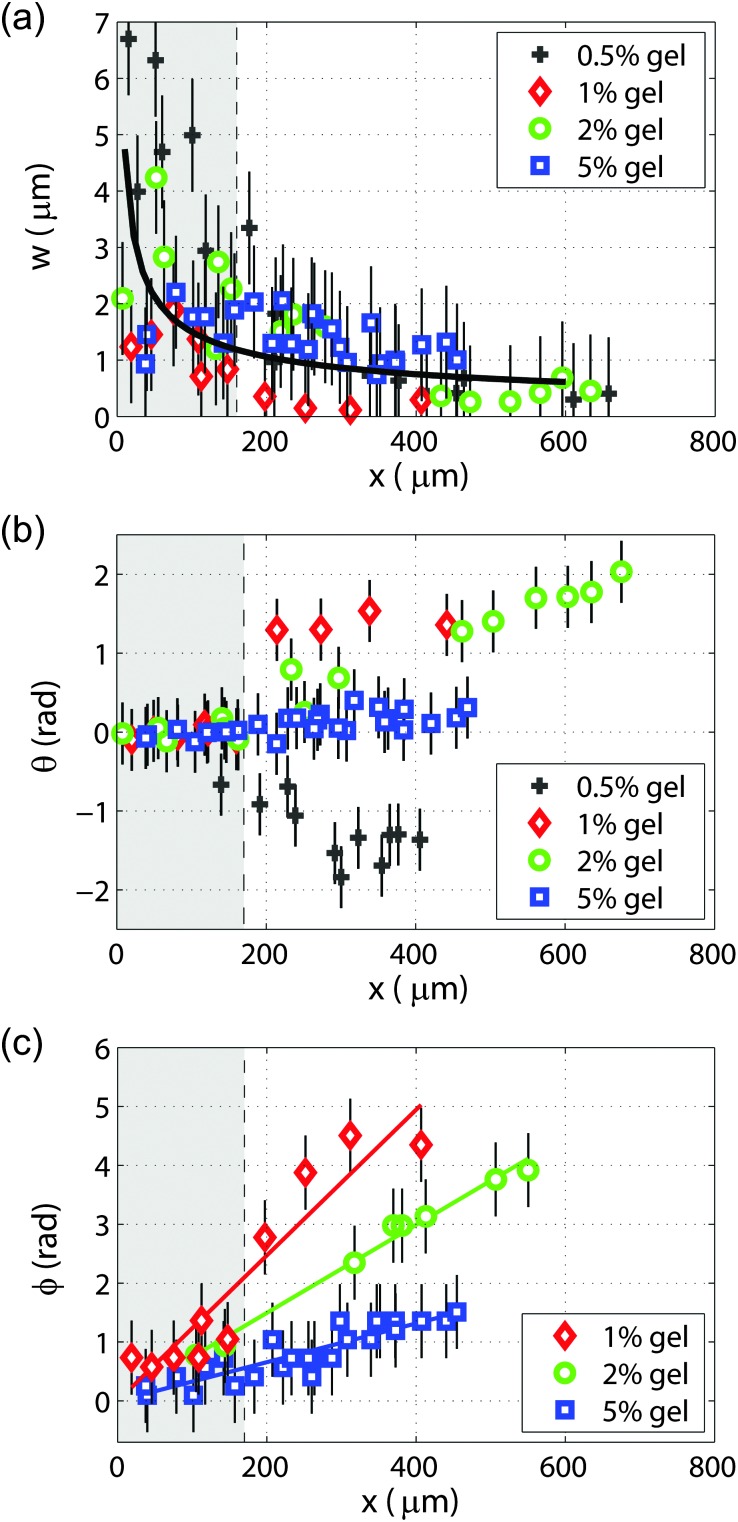
(a) Vertical particle displacement amplitude *w* as a function of distance from the bubble, for 0.5%, 1%, 2%, and 5% gels. The amplitude decay of cylindrical Rayleigh waves on a purely elastic half-space without viscous dissipation, scaling as *r* ^–1/2^, is shown for reference (solid line). The shaded area shows the radius of the bubble. (b) Tilt angle *θ* of the major axis of the elliptical trajectories as a function of distance for 0.5%, 1%, 2%, and 5% gel. (c) Phase shift *φ* between particle displacement and bubble oscillations as a function of distance for 1%, 2%, and 5% gel. The solid lines are linear fits used to extract the propagation velocity.

The variation with the distance from the bubble of the vertical amplitude of particle displacement, tilt angle of the elliptical trajectories, and phase shift between the particle displacements are shown in Fig. 4. The vertical particle displacement amplitude *w* as a function of distance from the bubble is plotted in Fig. 4(a) for the four gels. The amplitude decay of cylindrical Rayleigh waves on a purely elastic half-space without viscous dissipation,^32^ scaling as *r* ^–1/2^, is also shown for reference. Fig. 4(b) shows the variation of the tilt angle *θ* of the elliptical trajectories, measured when the maximum displacement occurs. The increase in tilt angle with distance from the bubble is larger for softer gels, and the tilt angle is negative for the softest gel (0.5%). The phase shift *φ* between the particle displacements as a function of distance is shown in Fig. 4(c). It was not possible to measure the phase shift as a function of distance with satisfactory accuracy for the 0.5% gel. The slope 
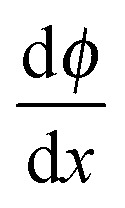
 of the linear fit to the experimental data, shown as a solid line in Fig. 4(c), is 
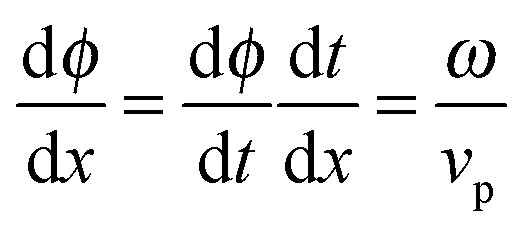
, where *ω* is the angular frequency and *v*_p_ the phase velocity of the propagating wave. We obtain values of the phase velocities *v*_p_ ≈ 11 m s^–1^ for the 1% gel, *v*_p_ ≈ 18 m s^–1^ for the 2% gel, and *v*_p_ ≈ 40 m s^–1^ for 5% gels.

## 4 Theory and modelling

The effect of the oscillating bubble on the gel can be modelled as a pressure applied to the surface of a viscoelastic solid. We assume that the deformations of the gel are small and, consequently, that the mechanics of the gel can be adequately described by the equations of linear viscoelasticity. In addition, the gel is considered to be a nearly incompressible Kelvin–Voigt body.^33^ The stress tensor can be decomposed into its elastic and viscous components *via* **T** = **T**_e_ + **T**_v_.

We consider a three-dimensional axisymmetric gel layer. The gel layer is assumed to occupy the region *r* > 0 and *z* > 0, where *r* = (*x* ^2^ + *y* ^2^)^1/2^ denotes the radial distance from the bubble-gel contact point located at *r* = 0 and *z* describes the vertical distance from the gel surface defined by *z* = 0; see Fig. 1. The model consists of a system of partial differential equations for the displacement vector **u**(*r*,*z*,*t*) = *u*(*r*,*z*,*t*)**e**_*r*_ + *w*(*r*,*z*,*t*)**e**_*z*_, where *t* is time; *u* and *w* are the radial and vertical components of the displacements, respectively; and **e**_*r*_ and **e**_*z*_ are unit vectors aligned with these directions.

The governing equations for the gel layer are given by

2a *ρ***ü** = ∇·(**T**_e_ + **T**_v_),

2b **T**_e_ = *λ*(∇·**u**)**I** + *G*(∇**u** + ∇**u**^T^),

2c **T**_v_ = *ξ*(∇·**u**)**I** + *η*(∇**u** + ∇**u**^T^),

where the dots denote differentiation with respect to time, *ρ* is the density of the gel, *λ* is the Lamé parameter, *G* is the shear modulus, and *ξ* and *η* are the dilational and shear viscosities, respectively. The boundary conditions at the gel surface are given by

3a **e**_*r*_·(**T**_e_ + **T**_v_)·**e**_*z*_ = 0, *z* = 0,

3b **e**_*z*_·(**T**_e_ + **T**_v_)·**e**_*z*_ = –*p*_b_(*r*,*t*), *z* = 0,

where *p*_b_ is the pressure applied by the oscillating bubble on the gel. Far away from the bubble, we assume that **u** → 0 due to the amplitude decay behaviour of cylindrical Rayleigh waves (see Fig. 4) and to viscous damping. Eqn (2a) describes conservation of linear momentum throughout the gel, while (3a) and (3b) represent the continuity of tangential and normal stress at the gel surface, respectively. Conservation of angular momentum requires the stress tensor to be symmetric, which is indeed the case. In deriving the boundary condition (3a), we have assumed that the bubble does not exert a shear stress on the surface of the viscoelastic gel; hence, continuity of stress requires that the tangential stress vanishes at *z* = 0. The pressure applied on the surface is modelled as Gaussian^34^ and sinusoidally oscillating:

4 *p*_b_(*r*,*t*) = *P*_0_e^–(*r*/*a*)^2^^ sin *ωt*

where *a* is the radius of the bubble, *ω* the angular frequency of the bubble oscillation, and *P*_0_ the maximum pressure at the contact point. The gel is initially at rest. Therefore, the initial conditions for the model are **u** = 0 and **u** = 0 at *t* = 0.

To gain insight into the nature of surface waves on a viscoelastic solid, we examine the behaviour of the model in the far field by writing5
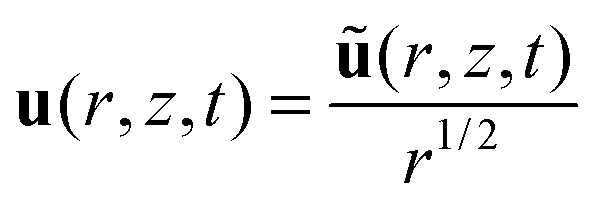
and then taking the limit as *r* → ∞. This limit has the effect of reducing the three-dimensional axisymmetric model to a two-dimensional Cartesian plane-strain model; that is, the radial coordinate *r* is essentially converted into a Cartesian coordinate in the far field. Surface waves on a two-dimensional Kelvin–Voigt viscoelastic layer have been studied in detail by ChiriŢă *et al.*,^35^ who show that viscosity leads to dispersive Rayleigh waves. Moreover, by seeking a travelling-wave solution to the governing equations of the form **ũ**(*r*,*z*,*t*) = **û**(*z*) exp[*ik*(*r* – *vt*)], ChiriŢă *et al.* are able to obtain a characteristic equation relating the wavenumber *k* to the complex wave speed *v* = Re(*v*) + *i* Im(*v*), where Re(*v*) > 0 and Im(*v*) ≤ 0 correspond to the phase speed and damping (in time) of the wave, respectively. In addition, the authors provide an analytical condition that can be used to determine whether the trajectories of surface particles are prograde or retrograde.

For a nearly incompressible medium as considered here, the characteristic equation for the complex wave speed reduces to the simple form6
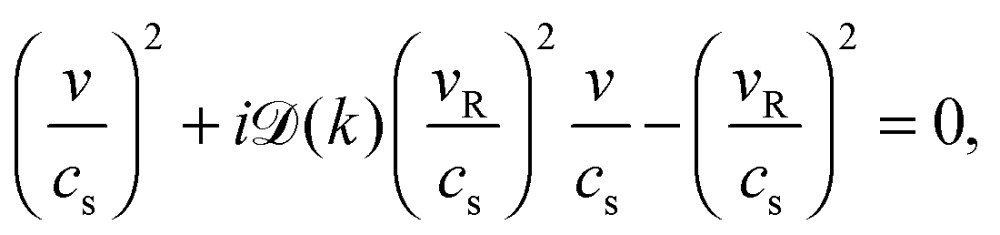
where *c*_s_ = (*G*/*ρ*)^1/2^ is the speed of a classical shear wave, *v*_R_ ≃ 0.955*c*_s_ is the speed of an incompressible elastic Rayleigh wave,^25,26^ and 𝒟 is a dimensionless wavenumber-dependent function given by

7 𝒟(*k*) = *kη*/(*ρc*_s_)

that leads to wave dispersion. If the dispersion function 𝒟 is small, which can occur in the weak-viscosity or long-wave limit, then the waves are non-dispersive and propagate with a speed given approximately by the elastic Rayleigh wave speed, *v* ≃ *v*_R_. Furthermore, the analysis predicts that particles on the surface of an incompressible body will always be retrograde. This suggests that the transition from prograde to retrograde motion is a near-field effect caused by the bubble, which we examine below *via* numerical simulation.

Since the wave speed has been experimentally measured, we can use (6) to calculate the shear modulus of the gels. We begin by estimating the order of magnitude of the dispersion function 𝒟 given by (7). The wavenumber of the experimental waves and the shear viscosity can be approximated using the relations *k* = *ω*/*v*_p_ and *η* = *G*′′/*ω*, respectively. From (6) we expect that *v*_p_ ∝ *c*_s_, which implies that 𝒟 ∝ *G*′′/*G*. Due to the fact that we were unable to measure the shear viscosity or loss modulus at the relevant frequency of deformation, 17–20 kHz, we make the key assumption that *G*′′ ≪ *G*′ and *G*′′ ≪ *G*. The assumption that the gel response remains predominantly elastic at high frequency is somewhat arbitrary, but it will be validated *a posteriori* from the comparison of the model predictions with experimental results. Under this assumption, we find that the dispersion function is small, 𝒟 ≪ 1; thus, the surface waves can be treated as non-dispersive Rayleigh waves that propagate with a velocity given by *v*_p_ = *v*_R_. Taking *ρ* ≃ 1000 kg m^–3^, we find for the 1%, 2% and 5% gels that *G* = 133 kPa, 355 kPa, and 1754 kPa, respectively. The high-frequency values of the shear modulus are an order of magnitude larger than those measured from the creep test (see Table 1). From these estimates of the shear modulus we find from eqn (1) crossover frequencies above 3 MHz for all gels, confirming that our observations are consistent with the propagation of Rayleigh waves.

The near-field wave dynamics are studied through numerical simulations. The numerical method is based on the explicit finite-difference scheme discussed in ref. 36 and 37 for a purely elastic medium. Due to issues relating to numerical stability, we found that it was more convenient to solve the problem in three-dimensional Cartesian coordinates rather than using an axisymmetric cylindrical coordinate system. The dimensions of the truncated computational domain were sufficiently large to prevent outgoing waves from reaching the boundaries and creating reflected waves. The number of free parameters in the numerical simulations is reduced by introducing dimensionless variables defined by *r*′ = *r*/*a*, *z*′ = *z*/*a*, *u*′ = *u*/*U*, *w*′ = *w*/*U*, and *t*′ = *ωt*, where *U* = *a*(*P*_0_/*G*)^1/2^ defines a scale for the displacement. The resulting model consists of four dimensionless parameters given by8
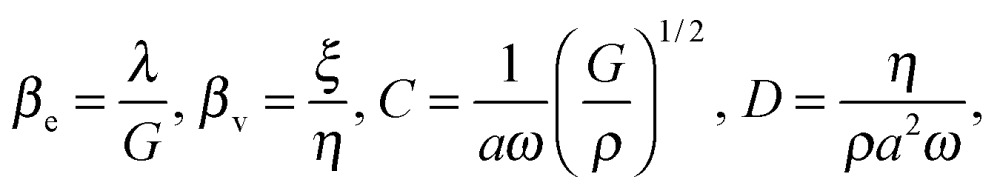
where *β*_e_ measures the compressibility of the gel, *β*_v_ characterises the relative viscous dissipation of longitudinal waves to shear waves, *C* is the non-dimensional speed of elastic shear waves, and *D* is the inverse Reynolds number. The parameter *β*_e_ can be related to the Poisson ratio of the gel *via β*_e_ = 2*ν*/(1 – 2*ν*). The assumption of near-incompressibility implies that *β*_e_ ≫ 1. The ratio *δ* = *D*/*C* ^2^ = (*η*/*G*)*ω* corresponds to the Deborah number, comparing the stress relaxation timescale, *η*/*G*, with the deformation timescale, *ω* ^–1^.

We fix *β*_e_ = 100, corresponding to a Poisson ratio of *ν* ≃ 0.495, and *β*_v_ = 0.1, since the dilational viscosity is expected to be much smaller than the shear viscosity.^38,39^ We investigate how the system responds to changes in *C* and *D*. In the parametric study we cover a range of values of *C* and *D* around those estimated from the wave propagation velocity. The angular frequency is *ω* = 113 × 10^3^ rad s^–1^, 107 × 10^3^ rad s^–1^, 126 × 10^3^ rad s^–1^, and 126 × 10^3^ rad s^–1^ for the 0.5%, 1%, 2% and 5% gels respectively. Taking *ρ* ≃ 1000 kg m^–3^, we find for the 1%, 2%, and 5% gels that *C* ≃ 0.57, 0.94, and 2.1 respectively. The value of *G* is not known for the 0.5% gel, but it is expected to be smaller than for the 1% gel. Estimating the value of *D* is non-trivial due to its dependence on the shear viscosity of the gel; however, in keeping with our previous assumption that the mechanical response of the gel remains elastic at high frequencies, we take *D* < *C*. To account for the uncertainties in these estimates of the viscoelastic properties, our computational study will cover the parameter space given by 0.05 < *C* < 2.1 and 0.01 < *D* < 0.5.

**Fig. 5:**
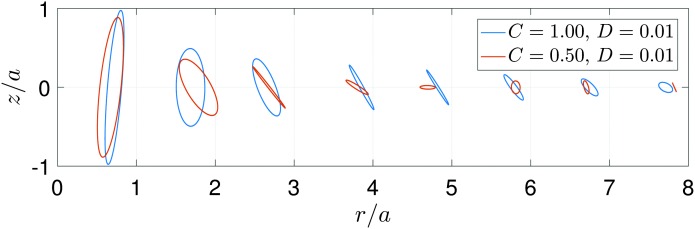
Particle trajectories on the surface of the gel obtained from the three-dimensional model of a Kelvin–Voigt viscoelastic solid, with *C* = 1 and *C* = 0.5, and *D* = 0.01. For clarity, the displacements are normalised by their maximum value.

**Fig. 6:**
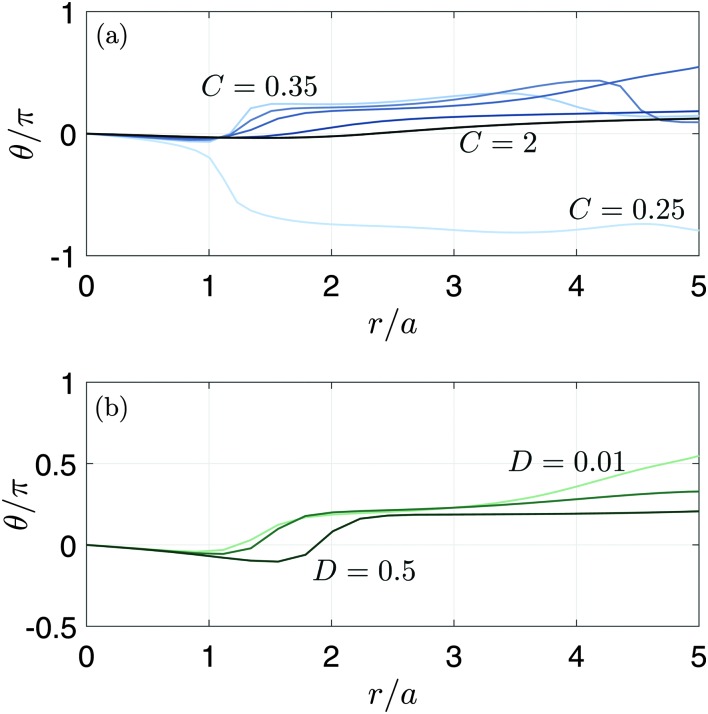
Tilt angle of the elliptical trajectories as a function of downstream distance from the flow origin, defined by *r* ^2^ = *x* ^2^ + *y* ^2^. This distance is normalised by the bubble radius *a*. (a) Parametric dependence on *C* with *D* fixed. We take *C* = 0.25, 0.35, 0.4, 0.5, 1 and 2 with *D* = 0.01. (b) Parametric dependence on *D* with *C* fixed. We take *D* = 0.01, 0.1 and 0.5 with *C* = 0.5.

Fig. 5 shows the trajectories obtained from the model predictions with *C* = 0.5 and *C* = 1, keeping *D* = 0.01. It is clearly seen that the trajectories are elliptical, with a tilt angle *θ* that varies with distance from the flow origin, in agreement with experiment. Decreasing *C* leads to stronger dampening of the surface waves, due to the increasing role of viscosity. The variation of the tilt angle with distance from the bubble is plotted in Fig. 6. For a fixed value of *D* = 0.01, we see that the spatial variation of the tilt angle depends strongly on the parameter *C* [Fig. 6(a)]. For soft or predominantly viscous gels, *C* ≤ 0.33 (*δ* ≥ 0.09), the tilt angle is always negative for distances up to five times the bubble radius. For stiffer or predominantly elastic gels, *C* > 0.33 (*δ* < 0.09), the tilt angle eventually becomes positive. The region of negative tilt angles that occurs near the bubble–gel contact point in Fig. 6(b) is likely an artifact of our simplified approach to modelling the effects of the bubble. Fig. 6(b) shows that, in contrast to *C*, the parameter *D* plays a relatively minor role in the spatial variation of the tilt angle for *D* ≤ 0.5 when *C* = 0.5. In this case, the curves remain qualitatively similar despite the Deborah number, *δ*, varying by two orders of magnitude, from 0.04 to 2. Since the parametric study reveals that *D* introduces no qualitative changes in spatio-temporal variations of *θ*, we will focus on the effect of *C* when comparing the model predictions with the experiments, with *D* fixed at 0.01.

## 5 Discussion

### 5.1 Comparison of experimental and modelling results

**Fig. 7:**
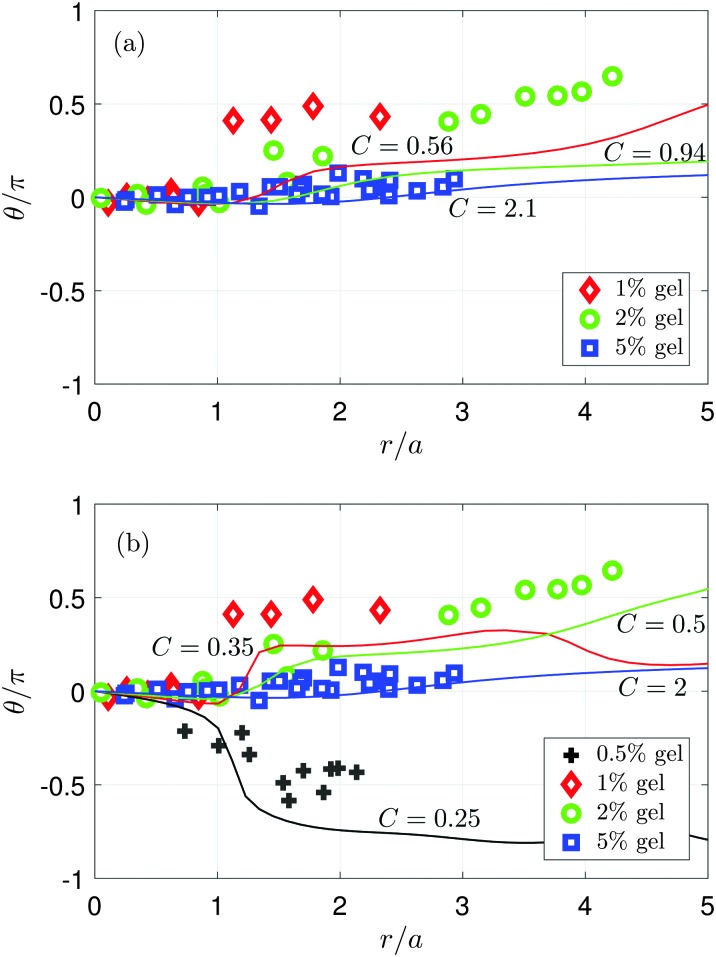
Tilt angle of the elliptical trajectories as a function of normalised distance from the flow origin. The symbols represent experimental measurements and the solid lines are computed from the model for various values of *C* and with *D* = 10^–2^. (a) Comparison of experiment with numerical simulations using values of *C* obtained from velocity measurements (see Table 2). (b) Comparison of experiment with simulation data over a wider range of *C* values. The model captures many of the qualitative trends seen experimentally.

Table 2 and Fig. 7 summarise the comparison of the model predictions with experimental results for the velocity of propagation of the surface wave, the location of the transition from prograde to retrograde particle motion, and the tilt angle variation with space. Using values of the shear modulus, *G*, that have been deduced from the velocity measurements, the model predicts wave speeds of 12 m s^–1^, 23 m s^–1^, and 44 m s^–1^ for the 1%, 2%, and 5% gels, respectively. The good agreement with the experimental values (11 m s^–1^, 18 m s^–1^, and 40 m s^–1^) supports the assumptions made in the model. The model predicts that the location of the transition from prograde to retrograde motion, measured relative to the radius of the bubble, is at 3.0, 4.2, and 6.5 for 1%, 2%, and 5% gels, respectively; the experimental values are 1.3, 2.2, and 4.3. Although the model appears to systematically overestimate the location of the transition, it does capture the fact that this quantity increases with the shear modulus.

**Table 2:** Characteristics of the surface deformation for 0.5%, 1%, 2%, and 5% gels. In the first set of data, the wave propagation velocity is experimentally measured and used to estimate values for the shear modulus *G* and hence the parameter *C*. The location of the transition from prograde to retrograde particle rotation, scaled by the bubble radius, is also reported. The second, third, and fourth sets of data are model predictions. In all cases, numerical simulations are used to estimate the wave propagation velocity and the location of the transition from pro- to retrograde rotation. In the second set of data, the shear modulus estimated from wave propagation velocity is used as input for the model. In the third set of data, the value of *C* is varied to obtain a closer fit to the experimental data. In the fourth set of data, the shear modulus measured from a creep test is used as input for the model

Gel concentration	0.5%	1%	2%	5%
Wave propagation velocity from experiment (m s^–1^)	N/A	11	18	40
Shear modulus *G* estimated from velocity (kPa)	N/A	133	355	1754
Non-dimensional shear wave speed *C* from experiment	N/A	0.57	0.94	2.1
Location of pro- to retrograde transition from experiment	1.2	1.3	2.2	4.3

Wave propagation velocity from model (m s^–1^)	N/A	12	23	44
Location of pro- to retrograde transition from model	N/A	3	4.2	6.5

Shear modulus *G* from fit (kPa)	26	50	101	1617
Non-dimensional shear wave speed *C*	0.25	0.35	0.5	2.0
Wave propagation velocity from fit (m s^–1^)	5.9	9.3	11	43
Location of pro- to retrograde transition from fit	N/A	5.6	2.9	6.4

Shear modulus *G* from creep test (kPa)	1.4	4.7	43	318
Non-dimensional shear wave speed *C*	0.06	0.11	0.33	0.89
Wave propagation velocity from model (m s^–1^)	N/A	N/A	8.8	22
Location of pro- to retrograde transition from model	N/A	N/A	7.9	4.1

Fig. 7(a) shows the variation of tilt angle (normalised by π) with distance that is obtained experimentally (symbols) and numerically (lines). The numerical curves are computed using values of *C* that are found from velocity measurements. We have not included the experimental results for the 0.5% gel, for which an estimate of *G* from the wave propagation velocity is not available. We see that there is reasonable agreement for the 5% (*C* = 2.1) gel, and although there are quantitative discrepancies for the 1% (*C* = 0.56) and 2% (*C* = 0.94) gels, the qualitative trend is correctly captured.

Fig. 7(b) highlights how the model can capture more closely the qualitative features of the experimental data as the parameter *C* is varied. The negative tilt angle far from the contact point is correctly predicted by the model when *C* ≤ 0.33. When *C* = 0.35, the model predicts a sharp increase in the tilt angle near *r*/*a* = 1, a feature that is seen experimentally in the 1% gel. The tilt angle for the 2% gel grows smoothly and steadily with distance from the contact point, which occurs in the model when *C* = 0.5. The tilt angle remains close to zero when *r*/*a* < 5 for the 5% gel and in simulations with *C* ≥ 2. The values of *G* obtained from the fit are 26 kPa, 50 kPa, 101 kPa, and 1617 kPa for the 0.5%, 1%, 2%, and 5% gel, respectively. The values of *G* are of the same order of magnitude as those found in experiment, and we have obtained an estimate of *G* for the 0.5% gel. The corresponding wave propagation velocities obtained from the fit are 5.9 m s^–1^, 9.3 m s^–1^, 11 m s^–1^, and 43 m s^–1^, in good agreement with the experimental values for the 1%, 2%, and 5% gels.

### 5.2 Effect of the high-frequency value of *G*

The model predictions are very sensitive to the value of *C*. In particular, using the properties measured at low frequency with a rheometer, one would obtain model predictions that are qualitatively incorrect. When using values of the shear modulus, *G*, from the creep test, we find that the corresponding values of *C* for the 0.5% and 1% gels are so low that it is not possible to accurately extract a wave speed from the model, or to locate the pro- to retrograde transition, because the material begins to behave as a fluid rather than an elastic solid. In the case of the 2% and 5% gels, the model predicts wave speeds of 8.8 m s^–1^ and 22 m s^–1^, respectively, which is roughly a factor of two smaller than the experimentally measured values of 18 m s^–1^ and 40 m s^–1^. In the case of a 2% gel, the model predicts a transition location that is nearly four times that which is observed experimentally, and negative tilt angles at all points along the gel surface, contrary to experimental observations (see Table 2).

### 5.3 Discussion of model assumptions

The small discrepancies between theory and experiments can be ascribed to the simplifications made in the derivation of the model; these include the assumption of small deformations, linear viscoelasticity, and the use of the Kelvin–Voigt approximation. Furthermore, the stress exerted on the gel by the oscillating bubble has been replaced in the model by a localised, oscillating pressure with constant amplitude in time and a Gaussian spatial decay, and the shear stress on the gel surface caused by the liquid around the bubble has not been taken into account. In addition, the vertical migration of the bubble slightly away from (towards) the gel during oscillations seen in experiment for the 0.5–2% (5%) gels has a concomitant effect on the pressure exerted by the bubble on the gel, and is not accounted for in the model. As the model captures the characteristic features of the deformation observed experimentally, and as there is no clear trend in the discrepancy between model and experiment for migration towards or away from the surface, these effects should be sub-dominant.

### 5.4 Transition from prograde to retrograde rotation

Rayleigh waves can exhibit both directions of rotation depending on the viscoelastic properties of the medium. In the particular case of an elastic medium, the rotation is expected to be retrograde, the major axis to be vertical, and the ellipticity *χ* = 0.56,^40^ *χ* representing the ratio between the horizontal and the vertical displacement of a particle during an oscillation period. However in our system we observe a transition from prograde to retrograde rotation for the same material. We explain this transition as a near-field effect due to the fact that the trajectories are probed in the vicinity of the contact point. Further from the bubble, we observed for both experimental and modelling results a retrograde rotation, which corresponds to the predictions found in the literature for our gel properties. Furthermore, far from the bubble (*r*′ > 25), the model predicts a constant vertical major axis, and an ellipticity *χ* ≈ 0.56, in agreement with the values from the literature. Elliptical particle trajectories have not been reported before for Rayleigh wave propagation on agarose gels. Because previous experiments used light scattering methods,^20,21,23^ only the surface profile could be detected. In contrast, by directly visualizing particle trajectories, we have reported both the component normal to the surface and the component parallel to the surface of the particle displacement.

## 6 Conclusion

We have performed time-resolved measurements of the deformation of a soft interface by oscillating microbubbles. We extracted the deformation of agarose gels by tracking with high-speed video microscopy the displacement of tracer particles embedded on the surface of the gel. We used four different gel compositions to study the effect of the rheological properties of the soft layer on the observed phenomena. We found that the localised deformation of the gel imparted by the bubble results in the propagation of surface elastic waves, or Rayleigh waves. During deformation, the tracer particles follow the characteristic elliptical trajectories observed upon propagation of a surface wave. Unexpectedly, we observed that the direction of rotation of the particles along the trajectories shifts from prograde to retrograde at a certain distance from the bubble. The tilt angle of the elliptical trajectories is also found to exhibit a strong dependence on the distance from the bubble. To explain these behaviours, we have developed a three-dimensional Kelvin–Voigt viscoelastic model for the deformation of gels by an oscillating force applied on the surface. The model predicts the occurrence of the phenomena observed experimentally, *i.e.*, the rate of change of the tilt angle of the elliptical particle trajectories, and the location of the transition from prograde to retrograde rotation. The model also captures the dependence of the response of the gel on its rheological properties. From the comparisons between experimental data and numerical simulations, we can conclude that the rheological properties of the gel measured at the relatively low frequencies attainable with conventional rheometers give qualitative disagreement between model and experiment. In contrast, the shear modulus at the frequency of the applied deformation, which in our experiments can be obtained from the velocity of the propagating Rayleigh wave, gives good qualitative agreement between model and experiment. Together, the experimental and modelling results presented should inform the development of predictive models of the effect of proximity of a viscoelastic boundary on bubble dynamics.
